# Establishment and validation of a nomogram for predicting overall survival of upper-tract urothelial carcinoma with bone metastasis: a population-based study

**DOI:** 10.1186/s12894-024-01488-7

**Published:** 2024-04-30

**Authors:** Jiasheng Hu, Haowen Gu, Dongxu Zhang, Min Wen, Zejun Yan, Baiyang Song, Chengxin Xie

**Affiliations:** 1 Department of Urology, The First Affiliated Hospital of Ningbo University, Ningbo, China; 2 Ningbo Clinical Research Center for Urological Disease, Ningbo, China; 3 Shandong First Medical University, Jinan, 250021 China; 4 Department of Orthopedics, Taizhou Hospital of Zhejiang Province Affiliated to Wenzhou Medical University, Linhai, China

**Keywords:** Upper-tract urothelial carcinoma, Bone metastasis, Prognosis, Nomograms, SEER program

## Abstract

**Background:**

Bone metastasis (BM) carries a poor prognosis for patients with upper-tract urothelial carcinoma (UTUC). This study aims to identify survival predictors and develop a prognostic nomogram for overall survival (OS) in UTUC patients with BM.

**Methods:**

The Surveillance, Epidemiology, and End Results database was used to select patients with UTUC between 2010 and 2019. The chi-square test was used to assess the baseline differences between the groups. Kaplan–Meier analysis was employed to assess OS. Univariate and multivariate analyses were conducted to identify prognostic factors for nomogram establishment. An independent cohort was used for external validation of the nomogram. The discrimination and calibration of the nomogram were evaluated using concordance index (C-index), area under receiver operating characteristic curve (AUC), calibration curve, and decision curve analysis (DCA). All statistical analyses were performed using SPSS 23.0 and R software 4.2.2.

**Results:**

The mean OS for UTUC patients with BM was 10 months (95% CI: 8.17 to 11.84), with 6-month OS, 1-year OS, and 3-year OS rates of 41%, 21%, and 3%, respectively. Multi-organ metastases (HR = 2.21, 95% CI: 1.66 to 2.95, *P* < 0.001), surgery (HR = 0.72, 95% CI: 0.56 to 0.91, *P* = 0.007), and chemotherapy (HR = 0.37, 95% CI: 0.3 to 0.46, *P* < 0.001) were identified as independent prognostic factors. The C-index was 0.725 for the training cohort and 0.854 for the validation cohort, and all AUC values were > 0.679. The calibration curve and DCA curve showed the accuracy and practicality of the nomogram.

**Conclusions:**

The OS of UTUC patients with BM was poor. Multi-organ metastases was a risk factor for OS, while surgery and chemotherapy were protective factors. Our nomogram was developed and validated to assist clinicians in evaluating the OS of UTUC patients with BM.

## Background

Upper-tract urothelial carcinoma (UTUC), specifically affecting the renal pelvis and ureter, represents a small proportion (5%-10%) of all urothelial carcinomas [1]. This carcinoma carries a poor prognosis, as approximately 60% of patients are diagnosed with invasive disease. A correlation has been observed between a higher T stage and a reduced 5-year survival rate [2]. Additionally, around 7% of individuals with UTUC present with distant metastasis, commonly in the lungs, liver, and bones, which significantly impacts cancer-related mortality [3]. Notably, bone metastasis (BM) in UTUC patients is associated with an unfavorable prognosis [4]. Therefore, thoroughly evaluating therapeutic strategies and prognostic factors is crucial to improving survival outcomes. Unfortunately, the literature on this topic is notably limited, with only a few retrospective studies available, all of which have small sample sizes.

Standard treatments for UTUC include radical nephroureterectomy, chemotherapy, and radiotherapy [5, 6]. However, there is a lack of research on the management of UTUC patients with BM. Recent studies suggest that surgery and chemotherapy are optimal approaches, but further investigation is necessary to validate these findings [7].

The Surveillance, Epidemiology, and End Results (SEER) Program, conducted by the National Cancer Institute (NCI), offers a comprehensive and widely accessible cancer database, regarded as one of the most extensive worldwide [8]. In tumor-related studies, the nomogram is a commonly used model that utilizes multivariate regression analysis to estimate a patient's likelihood of clinical events [9]. Currently, there is no prognostic model specifically developed for overall survival (OS) in UTUC patients with BM.

To address this gap, we utilized clinical data from the SEER database to characterize UTUC patients initially diagnosed with BM, identifying relevant prognostic factors systematically. Consequently, we developed a prognostic prediction model to assess the impact of each factor on the prognosis of UTUC patients with BM and predict OS.

## Methods

### Data source and patient selection

We utilized the case-listing session of SEER*Stat version 8.3.9 software (https://seer.cancer.gov/seerstat/) to extract clinical data from the SEER database for patients diagnosed with UTUC between 2010 and 2019. This time frame was chosen as sites of metastases were not recorded before 2010. The primary tumor sites of UTUC were selected using the International Classification of Diseases for Oncology, 3rd edition (ICD-O-3) codes "C65.9-Renal pelvis" and "C66.9-Ureter." The patients with BM were identified by designating "YES" in the column for "SEER Combined Mets at DX-bone (2010 +)". Patients with a confirmed pathological diagnosis and complete records of metastasis sites were included. All types of BM were taken into account, including solitary BM and BM combined with other metastatic sites. The distant metastasis events were recorded at the initial diagnosis of UTUC, and patients received treatment after diagnosis. Patients who meet any of the following criteria are excluded: aged under 18 years, unknown distant metastasis, prior malignancy, incomplete survival data, and unknown cause of death. We initially included all UTUC patients to investigate OS, but ultimately only patients with BM were selected for the construction and validation of the predictive model.

We established a validation cohort using electronic and/or papery medical records of UTUC patients with BM from The First Affiliated Hospital of Ningbo University diagnosed between April 1, 2003, and May 31, 2022. The criteria used to select the validation cohort are consistent with those applied to the training cohort. The study was approved by the licensing committee of The First Affiliated Hospital of Ningbo University (Approval NO.: 2023-163RS). Informed consent was waived by the Institutional Review Board of The First Affiliated Hospital of Ningbo University. All methods employed in this study adhered to relevant guidelines and regulations.

### Data collection and endpoint

We collected patients' clinical data, including age at diagnosis, sex, race, marital status, primary site, the total number of in situ/malignant tumors, laterality, histological type, T stage, N stage, tumor size, other metastases, surgery, radiotherapy, and chemotherapy. The primary endpoint was OS, defined as the time from diagnosis to death from any cause.

### Statistical analysis

The categorical variables were presented as frequency (percentage), and the chi-square test was used to assess the baseline differences between the groups. The mean and 95% confidence intervals (CIs) were used to describe the OS time. The OS was assessed using Kaplan–Meier analysis, and the survival curves were compared using the log-rank test.

Univariate and multivariate Cox regression models were employed to determine hazard ratios (HRs) and 95% CIs to identify independent prognostic factors. Baseline variables deemed clinically relevant or demonstrating a univariate relationship with the outcome were incorporated into the multivariate Cox proportional-hazards regression model. Variables for inclusion were carefully chosen, given the number of events available, to ensure parsimony of the final model [10]. Therefore, candidate variables with a *P* value less than 0.2 on univariate analysis were included in multivariable model [11].

Multivariable time-to-event analysis was performed using Cox proportional hazards regression models to develop a nomogram using weighted estimators corresponding to each covariate derived from fitted Cox regression coefficients and estimates of variance. Validation of the nomogram was evaluated by discrimination and calibration using the Harrell concordance index (C-index), area under receiver operating characteristic curve (AUC), calibration curve, and decision curve analysis (DCA) curve.

All statistical analyses and chart creation were conducted using SPSS 23.0 (IBM, Armonk, NY) and R software 4.2.2 (https://www.r-project.org/). A *P*-value less than 0.05 was considered statistically significant.

## Results

### Baseline characteristics

**Fig. 1:**
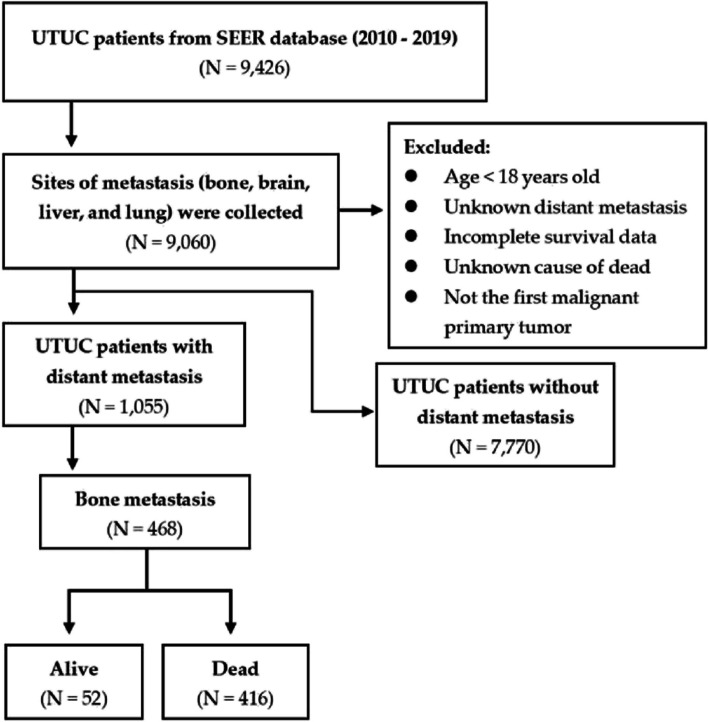
Flow chart of patient identification and selection from SEER

Based on the defined criteria, we initially identified 1,055 patients with distant metastasis and 7,770 patients without distant metastasis from the SEER database. Ultimately, a total of 468 patients with BM were included in the training cohort (Fig. 1). These comprised 212 patients (45.3%) with solitary BM, 5 patients (1.1%) with BM and brain metastasis, 78 patients (16.7%) with BM and liver metastasis, 96 patients (20.5%) with BM and lung metastasis, and 77 patients (16.5%) who had multiple metastases. In addition, 57 patients from The First Affiliated Hospital of Ningbo University were included in the validation cohort. The baseline characteristics of the patients from the two cohorts were compared in Table 1. Significant differences were observed in age (*P* = 0.035), race (*P* < 0.001), marital status (*P* < 0.001), tumor site (*P* < 0.001), TNT (*P* = 0.001), tumor laterality (*P* < 0.001), histological type (*P* < 0.001), T stage (*P* < 0.001), N stage (*P* < 0.001), tumor size (*P* < 0.001), surgery (*P* < 0.001), radiotherapy (*P* < 0.001), and chemotherapy (*P* = 0.039) between the training and validation sets.

**Table 1:** Baseline characteristics of UTUC patients with BM in the training cohort and validation cohort

	Training cohort	Validation cohort	*P* value
Variables	N (%)	N (%)	
Overall	468(100)	57(100)	
Age (years)			0.035
< 60	72(15.4)	5(8.8)	
60—79	277(59.2)	44(77.2)	
≥ 80	119(25.4)	8(14.0)	
Sex			1.000
Female	192((41.0)	23(40.4)	
Male	276(59.0)	34(59.6)	
Race			< 0.001
White	400(85.5)	0(0)	
Others	68(14.5)	57(100)	
Marital status			< 0.001
Married	249(53.2)	41(71.9)	
Single	68(14.5)	0	
Widowed	83(17.7)	16(28.1)	
Others	68(14.5)	0	
Primary site			< 0.001
Renal pelvis	344(73.5)	26(45.6)	
Ureter	124(26.5)	31(54.4)	
TNT			0.001
1	333(71.2)	51(89.5)	
≥ 2	135(28.8)	6(10.5)	
Laterality			< 0.001
Left	235(50.2)	0	
Right	224(47.9)	0	
Unknown	9(1.9)	57(100)	
Histological type			< 0.001
TCC	358(76.5)	57(100)	
Others	110(23.5)	0(0)	
T stage			< 0.001
T0–T2	88(18.8)	6(10.5)	
T3–T4	140(29.9)	2(3.5)	
TX	240(51.3)	49(86)	
N stage			< 0.001
N0	104(22.2)	8(14)	
N1	115(24.6)	0(0)	
N2–N3	142(30.3)	0(0)	
NX	107(22.9)	49(86)	
Tumor size (mm)			< 0.001
< 50	62(13.2)	30(52.6)	
≥ 50	79(16.9)	27(47.4)	
Unk	327(69.9)	0(0)	
Other metastases			0.263
None	212(45.3)	26(45.6)	
Brain	5(1.1)	3(5.3)	
Liver	78(16.7)	9(15.8)	
Lung	96(20.5)	10(17.5)	
Multiple	77(16.5)	9(15.8)	
Surgery			< 0.001
No/Unk	368(78.6)	29(50.9)	
Yes	100(21.4)	28(49.1)	
Radiotherapy			< 0.001
No/Unk	298(63.7)	57(100)	
Yes	170(36.3)	0(0)	
Chemotherapy			0.039
No/Unk	243(51.9)	22(38.6)	
Yes	225(48.1)	35(61.4)	

*TNT* Total number of in situ/malignant tumors, *TCC* Transitional cell carcinoma, *Unk* unknown

### Overall survival

**Fig. 2:**
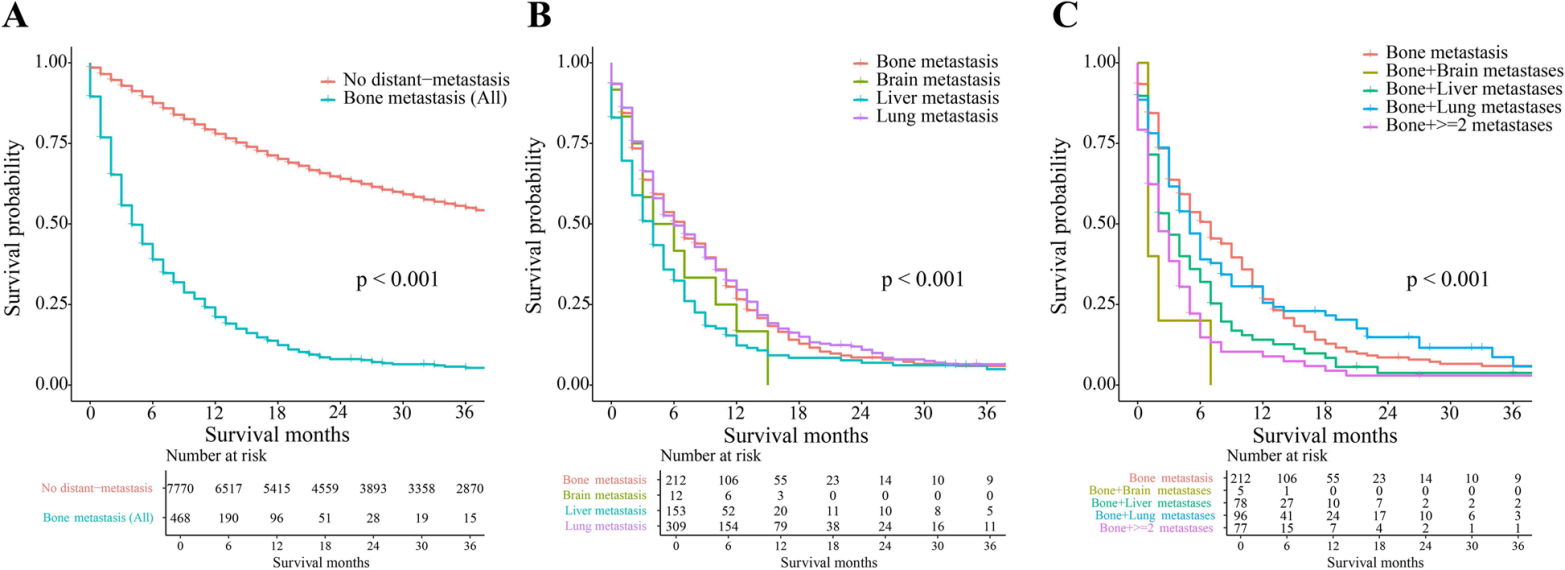
Kaplan–Meier survival curve of UTUC patients (**A** comparison of the OS between patients with BM and without distant metastasis; **B** comparison of the OS between patients with solitary BM and other types of solitary metastasis; **C** comparison of the OS between patients with solitary BM and multiple metastases)

The OS of 468 UTUC patients with BM was compared to that of 7,770 patients without distant metastasis. The mean OS for BM patients was 10 months (95%CI: 8.17 to 11.84 months), which was significantly shorter than 59 months (95%CI: 57.73 to 60.18 months) observed in patients without distant metastasis (Fig. 2A; *P* < 0.001). The 6-month OS, 1-year OS, and 3-year OS for BM patients were 41% (190/468), 21% (96/468), and 3% (15/468), respectively (Fig. 2A).

We conducted a subsequent analysis to compare the OS of UTUC patients with metastasis limited to a single site. Significant differences in OS were observed between patients with single metastasis in different sites (Fig. 2B; *P* < 0.001). Specifically, the OS of patients with BM (6-month OS: 50%, 1-year OS: 26%) was observed to be superior to those with liver metastasis (6-month OS: 34%, 1-year OS: 13%).

In addition, we compared the OS of UTUC patients with BM presenting in different metastasis patterns. A statistically significant difference in OS was observed among these patients with varied metastasis patterns (Fig. 2C; *P* < 0.001). The 6-month OS of patients with solitary BM was better than that of patients with both BM and other metastases.

### Prognostic factors for UTUC patients with BM

As shown in Table 2, the univariate analysis revealed that several factors were significantly associated with patients' OS. These candidate variables included age (*P* < 0.001), marital status (*P* = 0.07), TNT (*P* = 0.1), laterality (*P* = 0.1), presence of other metastases (*P* < 0.001), surgery (*P* = 0.001), radiotherapy (*P* = 0.1), and chemotherapy (*P* < 0.001). Multivariate Cox regression analysis identified the following factors as significantly associated with OS in BM patients: presence of multi-organ metastases (HR = 2.21, 95% CI: 1.66 to 2.95, *P* < 0.001), surgery performed (HR = 0.72, 95% CI: 0.56 to 0.91, *P* = 0.007), and chemotherapy performed (HR = 0.37, 95% CI: 0.3 to 0.46, *P* < 0.001).

**Table 2:** Univariate and multivariate Cox regression analysis of included variables for OS in training cohort

Variables	Univariable analysis	Multivariable analysis	
	*P* value	HR (95% CI)	*P* value
Age (years)	< 0.001		
< 60		1	
60—79		1.23(0.91 to 1.65)	0.173
≥ 80		1.24(0.88 to 1.75)	0.228
Sex	0.9	NP	
Race	0.2	NP	
Marital status	0.07		
Married		1	
Single		0.75(0.55 to 1.02)	0.065
Widowed		0.93(0.68 to 1.28)	0.665
Others		1.15(0.86 to 1.55)	0.343
Primary site	0.9	NP	
TNT	0.1		
1		1	
≥ 2		0.92(0.73 to 1.14)	0.44
Laterality	0.1		
Left		1	
Right		1.22(0.99 to 1.49)	0.058
Unknown		1.07(0.54 to 2.13)	0.838
Histological type	0.3	NP	
T stage	0.3	NP	
N stage	0.3	NP	
Tumor size (mm)	1	NP	
Other metastases	< 0.001		
None		1	
Brain		1.79(0.69 to 4.65)	0.229
Liver		1.31(0.99 to 1.72)	0.059
Lung		0.95(0.72 to 1.25)	0.705
Multiple		2.21(1.66 to 2.95)	< 0.001
Surgery	0.001		
No/Unk		1	
Yes		0.72(0.56 to 0.91)	0.007
Radiotherapy	0.1		
No/Unk		1	
Yes		0.9(0.73 to 1.11)	0.316
Chemotherapy	< 0.001		
No/Unk		1	
Yes		0.37(0.3 to 0.46)	< 0.001

*TNT* Total number of in situ/malignant tumors, *TCC* Transitional cell carcinoma, *Unk* Unknown, *NP* Not performed

### Construction of nomogram

**Fig. 3:**
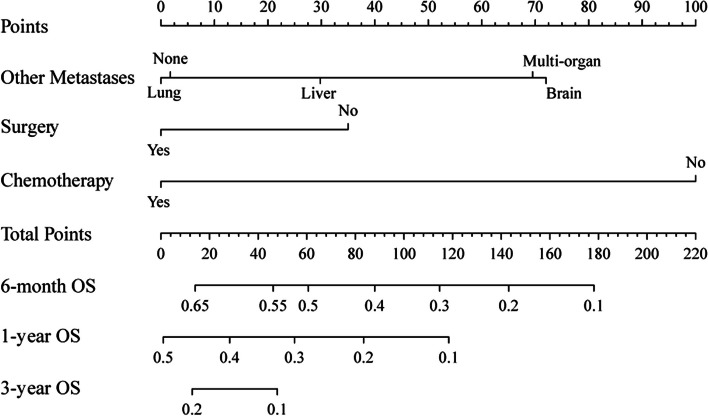
Nomogram for predicting OS of UTUC patients with BM

The nomogram was developed based on the results of the multivariate Cox regression analysis to predict the OS of UTUC patients with BM (Fig. 3). Each subgroup of the variables was assigned a score ranging from 0 to 100 on the top scale. By summing up the total score and locating it on the bottom scale, the estimated probability of survival at each time point (6 months, 1 year, and 3 years) can be easily determined by drawing a vertical line. The nomogram illustrated that chemotherapy had the most substantial contribution to the prognosis, followed by the presence of other metastases and surgery.

### Validation of nomogram

**Fig. 4:**
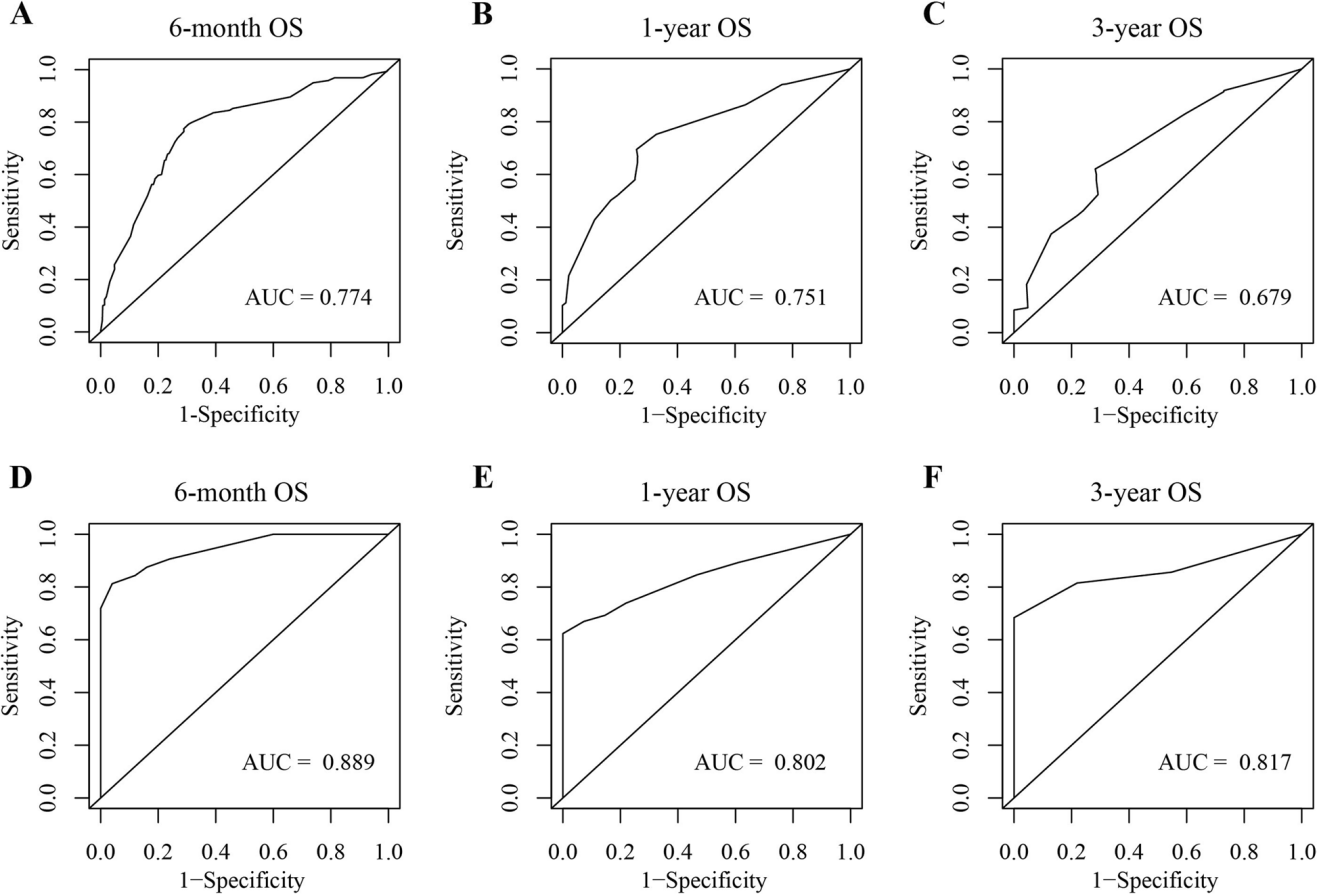
ROC curves of the prognostic nomogram (**A-C** training cohort; **D-F** validation cohort)

**Fig. 5:**
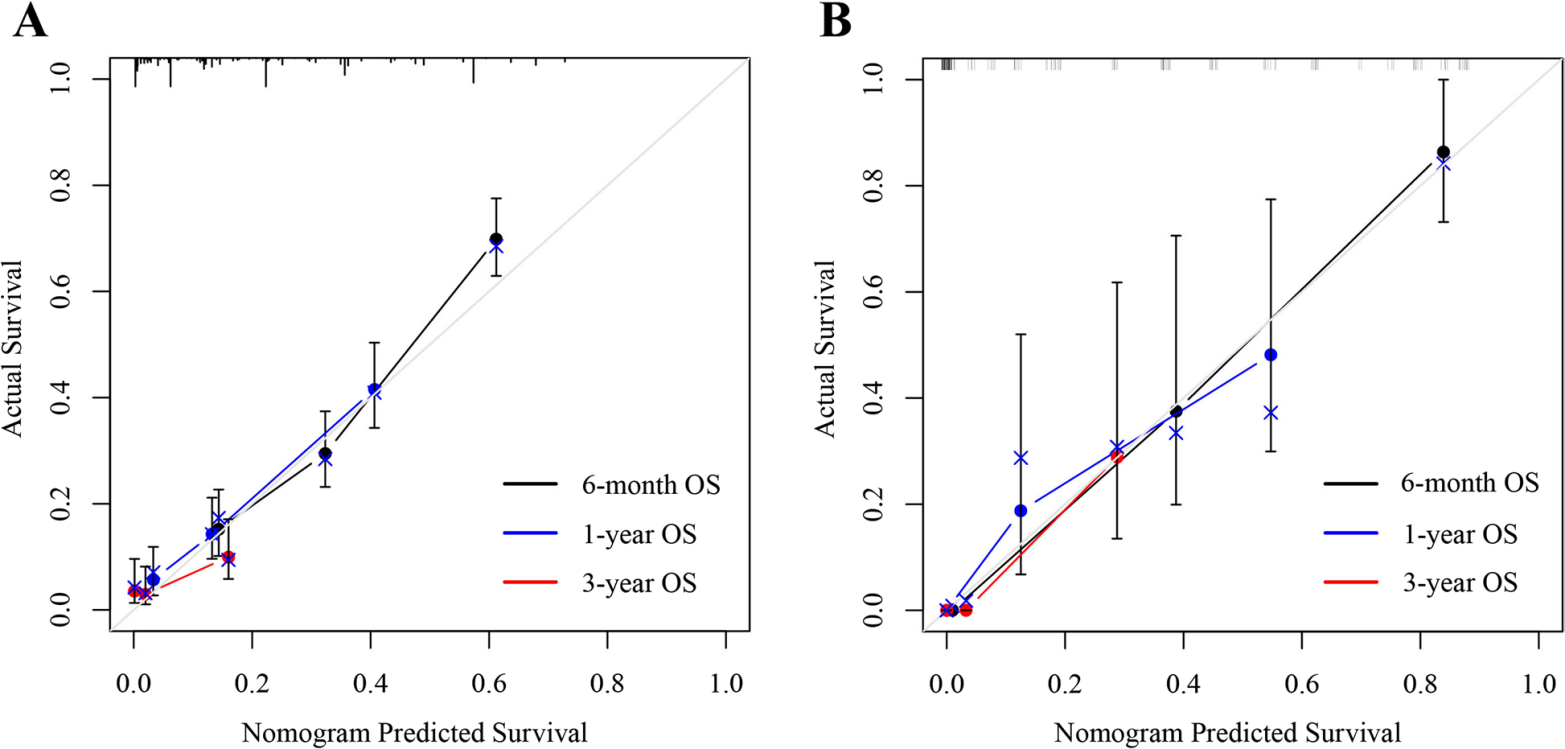
Calibration curve of the prognostic nomogram (**A** training cohort; **B** validation cohort)

**Fig. 6:**
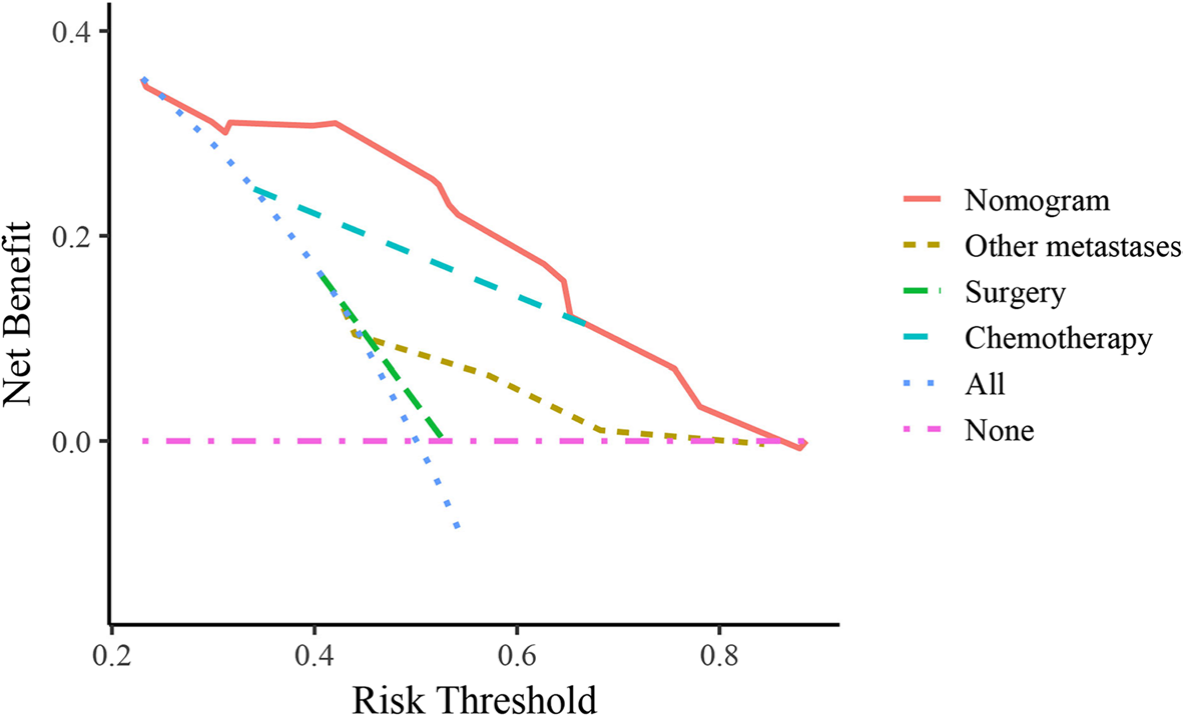
DCA curve of the prognostic nomogram

The C-index was 0.725 for the training cohort and 0.854 for the validation cohort. All AUC values were > 0.679 (Fig. 4). The calibration curve of the training cohort for 6-month, 1- and 3-year OS displayed an excellent fitting degree between the nomogram prediction and actual observation (Fig. 5A). Similarly, the calibration curve of the 6-month, 1- and 3-year OS were well calibrated in the validation cohort (Fig. 5B). The DCA curve graphically showed that the nomogram achieved the highest net benefit over a wide range (about 0.2 to 0.8) of reasonable threshold probabilities (Fig. 6).

## Discussion

UTUC with BM is a relatively rare subtype of urothelial carcinoma [12]. These patients have a poor prognosis, as shown by the survival curve in Fig. 2A, which demonstrates a more rapid decline compared to patients without distant metastasis over the next three years. Despite its rarity, the incidence of UTUC with BM has been increasing over the past few decades, highlighting the importance of exploring independent predictors of survival [13]. In this study, we conducted a comprehensive analysis of factors influencing the OS of UTUC patients with BM using the SEER database. Additionally, we developed a nomogram to assist clinicians and patients in making informed treatment decisions.

BM is a frequently observed site of metastasis in genitourinary cancer and has been the subject of extensive research. BM leads to skeletal-related events (SREs) such as pathological fractures, spinal cord compression, and hypercalcemia [14]. These events have a negative impact on the quality of life, increase healthcare burdens, and contribute to higher mortality rates. Early administration of bone-modifying agents to prevent SREs is recommended [15]. Owari et al. developed a scoring system specific to genitourinary cancer to predict survival rates in patients with BM, highlighting its clinical significance [16]. However, the limited set of predictors and a validation cohort with a lower proportion of patients undergoing surgery or radiotherapy make it challenging to draw definitive conclusions regarding treatment strategies.

Our study identified metastasis patterns, surgical interventions, and chemotherapy as key factors associated with OS in UTUC patients with BM, as determined by multivariate Cox regression analysis. Among these factors, chemotherapy had the most significant impact, followed by other metastases and surgery. Platinum-based chemotherapy (PBC) remains a crucial component of first-line treatment for metastatic urothelial carcinoma, extending median OS by three months [17]. Consistent with our findings, Alqaisi et al. reported improved OS in patients with BM after chemotherapy [18]. Additionally, chemotherapy facilitates the downstaging of UTUC, enabling surgical interventions [2, 19]. The combination of radical nephroureterectomy (RNU) and systemic chemotherapy for metastatic UTUC was found to be associated with an OS benefit compared to chemotherapy alone [20]. Furthermore, a retrospective study based on the National Cancer Database from the United States has demonstrated the high-intensity local treatments extended OS by 5 months in metastatic patients with stage IV UTUC [21]. Radiotherapy, as a routine treatment, can alleviate localized pain and improve SRE management [14, 22]. Maing et al. supported a radiation dose of ≥ 20 Gy as a factor in enhancing the prognosis of metastatic uroepithelial carcinoma [23]. However, Huang et al. found no significant survival benefit with radiotherapy in UTUC patients [24]. Therefore, we recommend an integrated approach involving surgery and chemotherapy as the optimal strategy for improving the prognosis of UTUC patients with BM.

The standard treatment for high-grade UTUC typically involves RNU and excision of the ipsilateral bladder cuff [25]. However, the significant rates of recurrence and cancer-specific mortality following surgery underscore the need for systemic treatments in the perioperative period to improve early-stage cancer management and patient survival [26]. Immunotherapy presents an opportunity to treat patients prior to RNU-related decline in renal function, potentially expanding the pool of patients eligible for PBC treatment by 30% [27]. In addition, for patients ineligible for chemotherapy, immunotherapy could represent a viable treatment option for metastatic UTUC [28]. Nonetheless, current data on the use of immunotherapy in managing metastatic UTUC is limited [25]. Considering the significance of chemotherapy in determining patient prognosis, immunotherapy may hold similar importance for patients who are ineligible for chemotherapy. Therefore, immunotherapy not only has the potential to enhance the effectiveness of chemotherapy and surgery but also holds promise as a novel alternative therapy.

Simultaneous metastases to other sites were common in UTUC patients with BM and significantly reduced their survival rates [7, 29]. Liu et al. suggested that the prognosis of stage IV metastatic uroepithelial carcinoma depends on the number of metastases from other organs, with no difference in OS compared to other metastatic sites when using bone metastasis as a reference [30]. Importantly, our findings contrast with those of Zhou et al. [7], who reported that lung metastasis was identified as a risk factor for OS in UTUC patients with BM. This discrepancy could be attributed to the fact that their study did not further stratify cases based on the presence of multiple metastases. It is plausible that the presence of metastasis in other regions along with lung metastasis contributes to a poorer OS, rather than lung metastasis alone being the sole causative factor. In addition, their study did not develop a nomogram to facilitate clinical decision-making [7]. Our research addresses these gaps and indicates that multi-organ metastases are independent risk factors.

To the best of our knowledge, this is the first nomogram developed to predict the survival of UTUC patients with BM based on a large database with long-term follow-up. Importantly, we reviewed hospitalization medical records from a single center over the past 20 years to collect patients and establish an external validation set. However, several limitations of our study should be taken into account. Firstly, it is important to acknowledge that our study is retrospective in nature, which may introduce selection bias. This bias arises from the fact that patients who received more aggressive therapies may have inherently better chances of survival. Patients who did not receive chemotherapy were likely individuals with a notably unfavorable prognosis, potentially due to the presence of comorbidities. Secondly, the database lacked some potential prognostic parameters, such as smoking status, performance status, comorbidities, and time to metastasis. Thirdly, the database did not provide specific details on surgical modalities, radiotherapy regimens, chemotherapy protocols, and systemic treatments. Fourthly, the patients who develop new tumors after being diagnosed with BM were not excluded. Fifthly, surgery information recorded in the SEER database typically focuses on the primary site, and not all patients undergo surgery for metastatic sites. Additionally, the number of cases involving relevant treatments like radiotherapy was minimal. Lastly, it should be noted that our single-center external dataset lacks representativeness, as there is a substantial difference in population characteristics compared to the training group.

## Conclusions

In conclusion, the OS of UTUC patients with BM is worse than that of patients without distant metastases. The OS of patients with solitary BM is better than that of patients with solitary liver metastasis. Multi-organ metastases are independent risk factors for OS in patients with BM, while surgery and chemotherapy are protective factors. Our nomogram, which incorporates metastasis patterns, surgery, and chemotherapy, is expected to serve as an individualized tool for clinicians to estimate the OS of UTUC patients with BM.

## Data Availability

The datasets generated and/or analyzed during the current study are available in the SEER database, https://seer.cancer.gov/. Other datasets are available from the corresponding author on reasonable request.

## Abbreviations

BM: Bone metastasis
UTUC: Upper-tract urothelial carcinoma
OS: Overall survival
AUC: Area under receiver operating characteristic curve
DCA: Decision curve analysis
SEER: Surveillance, Epidemiology, and End Results
NCI: National Cancer Institute
HR: Hazard ratio
CI: Confidence interval
SRE: Skeletal-related event
PBC: Platinum-based chemotherapy
RNU: Radical nephroureterectomy
